# Can the neural representation of physical pain predict empathy for pain in others?

**DOI:** 10.1093/scan/nsae023

**Published:** 2024-03-14

**Authors:** M Li, C Racey, C L Rae, W Strawson, H D Critchley, J Ward

**Affiliations:** School of Psychology, University of Sussex, Brighton BN1 9QH, UK; School of Psychology, University of Sussex, Brighton BN1 9QH, UK; School of Psychology, University of Sussex, Brighton BN1 9QH, UK; Brighton and Sussex Medical School, University of Sussex, Brighton BN1 9PX, UK; Brighton and Sussex Medical School, University of Sussex, Brighton BN1 9PX, UK; School of Psychology, University of Sussex, Brighton BN1 9QH, UK

**Keywords:** pain, empathy, biomarker, fMRI, individual differences

## Abstract

The question of whether physical pain and vicarious pain have some shared neural substrates is unresolved. Recent research has argued that physical and vicarious pain are represented by dissociable multivariate brain patterns by creating biomarkers for physical pain (Neurologic Pain Signature, NPS) and vicarious pain (Vicarious Pain Signature, VPS), respectively. In the current research, the NPS and two versions of the VPS were applied to three fMRI datasets (one new, two published) relating to vicarious pain which focused on between-subject differences in vicarious pain (Datasets 1 and 3) and within-subject manipulations of perspective taking (Dataset 2). Results show that (i) NPS can distinguish brain responses to images of pain *vs* no-pain and to a greater extent in vicarious pain responders who report experiencing pain when observing pain and (ii) neither version of the VPS mapped on to individual differences in vicarious pain and the two versions differed in their success in predicting vicarious pain overall. This study suggests that the NPS (created to detect physical pain) is, under some circumstances, sensitive to vicarious pain and there is significant variability in VPS measures (created to detect vicarious pain) to act as generalizable biomarkers of vicarious pain.

## Introduction

Vicarious pain may involve an internal simulation (or mirroring) of pain that uses some of the same neural resources that process physical pain (as assessed by neuroimaging methods). The majority of previous studies used univariate analysis of fMRI data which are based on the overlapping brain activations when experiencing pain and seeing others in pain (Lamm *et al*., 2011; Xiang *et al*., 2018). Although this method has proven effective for inference in traditional localization analysis (Friston *et al*., 1995), they are less appropriate for estimating the precise information encoded in multiple voxels that underlay a certain cognitive process and this has led some to doubt whether vicarious pain and physical pain overlap at the voxel-level of granularity (Krishnan *et al*., 2016).

Over the past two decades, multivariate pattern analysis (MVPA) has become a key research technique in task-evoked neuroimaging studies (Norman *et al*., 2006; Haxby, 2012). MVPA considers neural responses as patterns of brain activity entered into a single analysis (machine learning), rather than entering each voxel into multiple (univariate) analyses (Norman *et al*., 2006; Mahmoudi *et al*., 2012). Whereas traditional univariate approaches measure the difference between conditions (e.g. mean activity), the aim of MVPA is to determine if patterns of activity predict performance above chance; for example, to determine whether a participant is viewing a face or a house (a categorical prediction or ‘classifier’) or to determine the degree of pain they are in (on a continuous scale). This approach typically uses two sets of data: a training set which is used to develop the algorithm and a statistically independent test set (which provides the basis for calculating predictive accuracy). Training and test data are normally created by iteratively splitting a single dataset (termed k-fold cross-validation). This is often done within an individual subject but in theory, one can also develop a classifier for one set of people (e.g. on a scanner in the USA) and test them on a different group of people in different conditions (e.g. on a scanner in Europe). This is an aim for the development of brain-based biomarkers for clinical conditions (e.g. dementia) and clinical symptoms (e.g. pain) (Arbabshirani *et al*., 2017; Woo *et al*., 2017) which, ideally, would generalize across contexts. Whereas MVPA approaches for studying cognition often focus on specific regions (e.g. insular cortex), clinical biomarkers tend to be based on the whole brain or multiple brain regions. Here, we take putative biomarkers developed for physical and vicarious pain, developed and published by others, and apply them to three datasets acquired locally to understand whether such biomarkers generalize across individual differences in vicarious pain and task instructions.

Wager *et al.* (2013) developed a potential biomarker for physical pain that they term the Neurologic Pain Signature (NPS). Thermal pain stimulation at four levels (one of which was innocuous warmth) was delivered to participants and the aim was to be able to predict the level of pain in one person from the averaged pattern of brain activity in the remaining sample. Multiple candidate brain regions were selected as having been identified as related to pain by previous research (using the NeuroSynth meta-analytic database; Yarkoni *et al*., 2011) and the voxel-level pattern of activity within this mask was entered into a classifier. The classifier was able to discriminate thermal pain from non-painful warmth above 90% and was replicable across scanners. The NPS, although designed to detect thermal pain, can accurately detect other forms of acute physical pain, including visceral distention (Woo *et al*., 2017), mechanical pressure (Krishnan *et al*., 2016) and electric shock (Ma *et al*., 2016). It also reflects pain modulation by opioids and serotonergic drugs (Wager *et al.*, 2013).

One key claim about the NPS is that it does not predict either social or vicarious ‘pain’. Wager *et al.* (2013) applied the NPS to fMRI activation maps from a previous study that compared brain activity when viewing images of a romantic partner who had jilted them (social pain) *vs* images of a friend (Kross *et al*., 2011). Notably, the original univariate analysis showed that a simple contrast between these conditions activated regions of the pain matrix (insula, anterior cingulate). However, the NPS showed chance-level discrimination (Woo *et al*., 2014). With regard to classifying emotional intensity of International Affective Picture System (IAPS) images, the NPS could accurately classify physical pain from emotional arousal (Chang *et al*., 2015). The NPS response could also classify, significantly above chance, different levels of emotional intensity amongst IAPS images (but didn’t show a monotonic trend from low, medium and high emotion intensity). Finally, Krishnan *et al*. (2016) applied the NPS to vicarious pain. Specifically, participants were shown images of hands and feet in pain (e.g. trapped in a door) and asked to engage in perspective taking (imagine the injuries were happening to them) and then rate the intensity. The NPS was not able to discriminate high *vs* low ratings of vicarious pain but produced consistently higher responses for physical *vs* vicarious pain (and could discriminate high *vs* low physical pain). They also developed a brain biomarker that was sensitive to varying levels of vicarious pain, termed the VPS (Vicarious Pain Signature), which incorporated regions such as those involved in mentalizing (e.g. temporo-parietal junction). This could not predict high *vs* low physical pain (but could predict high *vs* low vicarious pain). On this basis, they claimed that there are no shared representations between physical and vicarious pain.

It is important to note that not all previous research using MVPA-based approaches support this conclusion. Corradi-Dell’Acqua *et al*. (2011) contrasted physical pain (thermal pain to the hand) and images of the hand either in pain or not in pain. For the latter, participants were asked to identify the handedness of the hand, i.e. vicarious pain was incidental. A region in the right insula was able to cross-classify vicarious pain (from a classifier trained to discriminate physical pain) and was similarly able to cross-classify physical pain (from a classifier trained to discriminate pain *vs* no-pain in the images). Similar findings were reported in a follow-up study that compared physical pain and disgust with vicarious pain and disgust (Corradi-Dell’Acqua *et al*., 2016). Zhou *et al*. (2020) developed an alternative version of the VPS by taking images of limbs in pain *vs* no-pain and facial expressions of pain and no-pain. There was no task inside the scanner (i.e. passive viewing), and MVPA classifiers were developed to distinguish these pairs of conditions. In addition, they created a generalized classifier trained to distinguish the two pain conditions from their two corresponding control conditions. This generalized classifier (here termed VPS-Zhou) was able to accurately distinguish different levels of thermal pain from the original NPS dataset (Wager *et al*., 2013) using two-alternative forced choice. This also extended to a mid-insula region of interest. These results support the idea of shared representations between physical and vicarious pain.

As such, further research is needed to understand the conditions (if any) in which vicarious and physical pain use shared neural resources at the level of voxel-based patterns of activity. Differences may be due to the task, the stimuli or the participants themselves. For example, Corradi-Dell’Acqua *et al*. (2011) contrasted images of pain *vs* no-pain (a categorical distinction) whereas Krishnan *et al*. (2016) used only painful images (without any no-pain counterparts) that were subjectively rated on a continuous scale. There are stronger tendencies towards vicarious pain in East Asian populations (Li *et al*., 2022), which may affect the generalizability of biomarkers across cultures. Moreover, there may be individual differences in the extent to which vicarious pain resembles physical pain. Grice-Jackson *et al*. (2017a) developed the VPQ (Vicarious Pain Questionnaire) in which videos of pain (e.g. an injection, a bike fall) are rated in various ways (whether they elicit pain in the observed and, if so, the qualities of that pain including its localization). A cluster analysis found three groups: most people (∼75%) do not experience vicarious pain when seeing others in pain (non-responder group), and those who experience vicarious pain can be divided into affective/general (A/G) group (who tend to report affective and non-localized pain) and sensory/localizer (S/L) group (who tend to report sensory and localized pain). In a univariate fMRI analysis, contrasting images of pain against no-pain, the A/G and S/L group showed increased activity in regions linked to physical pain (Grice-Jackson *et al*., 2017b). No physical pain was presented to the participants and so this precluded a conventional MVPA analysis in which a classifier developed for physical pain is applied to vicarious pain. However, a less conventional approach would be to apply the NPS biomarker to this and similar datasets.

This study applies both the NPS (Wager *et al*., 2013) and two versions of the VPS (Krishnan *et al*., 2016; Zhou *et al*., 2020) to three datasets that have used a standard vicarious pain manipulation of showing images of hands or feet that are either in pain or not in pain (using the same set of images created by Jackson *et al*., 2005). Based on the conclusions of Krishnan *et al*. (2016), the prediction is that the VPS, but not the NPS, would be able to categorize patterns of brain activity when observing images of pain *vs* no-pain. Based on the findings of Grice-Jackson *et al*. (2017b), we would predict that the NPS would be better able to classify patterns of brain activity linked to vicarious pain in the responder groups. Dataset 1 is from Grice-Jackson *et al*. (2017b), with participants divided into three groups (non-responders, S/L and A/G), and in which they were asked to rate their own vicarious pain response to seeing painful and non-painful images. Dataset 2 uses a sample of students from Rae *et al*. (2020) who we would assume would be primarily non-responders. They were shown images of hands and feet (pain and no-pain) with two sets of instructions: self-perspective or other perspective. By including this study, we can explore the extent to which these pain signatures are sensitive to task instructions. Dataset 3 again contrasted vicarious pain responders (S/L and A/G) and non-responders but when passively viewing this same set of images. This last dataset has not previously been published.

## Methods

**Fig. 1.**
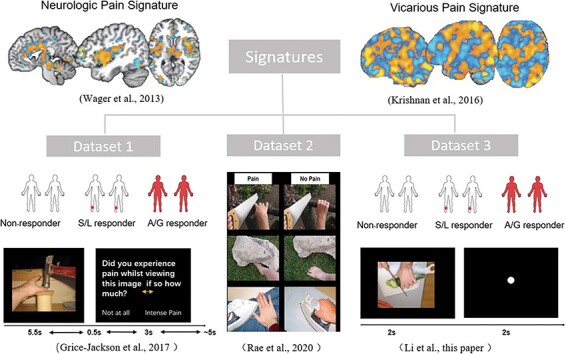
The flow chart of the experiment design. Existing signatures (neurologic pain signature, NPS and two vicarious pain signatures, VPS) were applied to three independent datasets (only one version of the VPS is shown).

An overview is shown in Figure 1. All studies make use of the pain and no-pain images of hands and feet that have been commonly used in this field (Jackson *et al*., 2005), albeit with different participant groups and different task instructions as detailed below.

### Dataset 1: reanalysis of Grice-Jackson *et al.*, 2017b

There were 40 healthy participants in dataset 1 which included 18 non-responders, 12 S/L responders and 10 A/G responders (note the original dataset contained four additional participants, but the data were missing for reanalysis), with group classification based on the Vicarious Pain Questionnaire (VPQ) (Grice-Jackson *et al*., 2017a). All 40 participants finished an image-based empathy task. This task is a 2 (pain levels: pain and no-pain) × 2 (topography: hand and foot) event-related design. Each experimental trial began with viewing a picture (5.5 s) followed by a blank screen (0.5 s). Participants then had to make a rating of the pain felt on their own body. Details of pre-processing, first-level general linear model (GLM) analyses and more detailed design information can be found in the work by Grice-Jackson *et al.* (2017b). The relevant data consists of 40 × 2 voxel-based beta maps, where each participant has a separate map corresponding to viewing pain and no-pain images.

### Dataset 2: reanalysis of Rae et al. (2020)

Seventy-one participants finished an image-based empathy task with both self and other’s perspective (Rae *et al*., 2020). They were recruited on the basis of different levels of social alcohol drinking, but that variable was not of interest here. This task consisted of four conditions in a 2 × 2 design: in which pain and no-pain images were viewed under different task instructions (self or other perspective). At the start of each block, the participant was instructed by a 30-s written cue to adopt the perspective that the images depicted events occurring either to themselves (Self condition) or to another, unfamiliar, person (Other condition). No physical pain was present during the task (so ‘Self’, in this context, refers only to the perspective taken). Each experimental trial began with viewing a picture (2 s) and subjects were required to press a key to indicate whether the image depicted a painful scene or a non-painful scene. The image was followed by a fixation (2 s). Details of pre-processing, first-level general linear model (GLM) analyses and more detailed design information can be found in Rae *et al*. (2020). The relevant data consists of 71 × 4 voxel-based beta maps, where each participant has four separate maps corresponding to viewing pain and no-pain images during the self and other conditions.

### Dataset 3: novel imaging study on individual differences

As this study is based on unpublished data, it is described in detail.

#### Participants

There were 64 healthy participants in dataset 3 recruited from the student population of the University of Sussex, UK, which included 25 non-responders (mean age = 20.28, SD =2.36, male = 8), 19 S/L responders (mean age = 22.60, SD =6.85, male = 5) and 20 A/G responders (mean age = 21.45, SD =3.15, male = 4), with group classification based on the VPQ (Grice-Jackson *et al*., 2017a) with clusters extracted from a larger group (Ward and Li, 2022). All the subjects were provided informed consent at the beginning of the study, and they were paid £20 for their participation. This study was reviewed and approved by the Brighton and Sussex Medical School (BSMS) Research Governance and Ethics Committee of the University of Sussex.

#### Materials and task procedure

All 64 participants finished a picture-based empathy task. This task is a 2 (foot; hand) × 2 (no-pain; pain) design, which included a series of 128 images showing hands and feet experiencing different types of pain with contextual matched no-pain images (the same as study 1). Each experimental trial began with viewing a picture (2 s) followed by a white circle (2 s). The participants were not required to rate them. There were 10 blank trials (4 s) that were unmodelled and acted as part of the baseline.

#### MRI data acquisition

A 3T Siemens Prisma scanner with a 64-channel head-coil was used to acquire all images. Functional images were acquired using the Human Connectome Project (HCP) gradient-echo EPI sequence, with a multiband acceleration factor of 8; TR = 0.8 s; TE = 33.1 ms; 52 degree flip angle; FOV = 208 × 180 mm; 72 slices with slice thickness of 2 mm and isotropic 2 mm voxels. Two SpinEcho Field maps with reversed phase-encode blips in both Anterior to Posterior and Posterior to Anterior were acquired with the same parameters as the functional images. A high-resolution structural T1-weighted image was acquired with 3D MPRAGE sequence (TR = 2.4 s; TE = 2.14 s; 8 degree flip angle; FOV = 224 × 224 mm and 0.8 mm isotropic voxels).

#### MRI data pre-processing

Pre-processing was performed using a mix of FMRIB Software Library (FSL) and Statistical Parametric Mapping (SPM12) tools. T1-weighted anatomical volumes were corrected for gradient non-linearities based on scanner calibration measurements. Non-brain data were removed from each T1 volume using the FMRIB Brain Extraction Tool (Smith, 2002; Jenkinson *et al*., 2005), then aligned to the Montreal Neurological Institute (MNI) 152 standard template anatomical image using FSL’s FMRIB’s Linear Image Registration Tool with 12 degrees of freedom (Jenkinson and Smith, 2001; Jenkinson *et al*., 2002). Each volume was inspected for image artefacts and tissue contrast and rejected if deemed of poor quality.

Both temporal and spatial pre-processing were performed on the fMRI data. Field maps were estimated and applied using FSL’s top-up utility to correct for image distortions (Andersson *et al*., 2001). Cubic interpolation was performed on each voxel’s time-series data to correct for differences in slice acquisition times and to obtain an integer sampling rate of 1.0 s. Rigid-body motion parameters were estimated from the undistorted EPI volumes using the SPM12 utility spm_realign. Finally, cubic interpolation was performed on each slice time-corrected volume to compensate for the combined effects of EPI distortion and motion. No spatial smoothing or temporal filtering was performed. The average of the pre-processed functional volumes was coregistered to the T1 anatomical volume (affine transformation estimated using a 3D ellipsoid that focuses the cost metric on cortical tissue). This resulted in a transformation that maps the EPI data to the participant-native brain anatomy volume.

Data scrubbing was also implemented to address head motion issues. No participant was excluded from further analyses because of excessive head motion. To compare the effects of head motion among different groups, we calculated framewise displacement (FD) head motion for each task. Framewise head motion calculates the relative head motion of each timepoint to its prior timepoint (Power *et al*., 2014). One-way ANOVA was performed on FD among the three groups. Results showed there was no significant difference among three groups in the task [F(2, 63) = 0.661, *P* = 0.520].

Image presentation was modelled in SPM using regressors (for pain and no-pain) that convolved with the canonical hemodynamic response function and blank trials that remained served as an implicit baseline. GLM-denoise was applied (available MATLAB code at http://kendrickkay.net/GLMdenoise/) which derives the optimal number of principle components to use as noise regressors (Kay *et al*., 2013). The relevant data consist of 64 × 2 voxel-based beta maps, where each participant has a separate map corresponding to viewing pain and no-pain images.

### Analysis: the neurologic and vicarious pain signatures

Neurologic Pain Signature (NPS) is a multivariate brain model designed to make predictions of physical pain (Wager *et al.*, 2013). Namely, it is a brain map that includes weights in each voxel of a defined brain mask of pain-related regions. For example, negative weights represent these voxels contribute negatively in predicting pain intensity while positive weights represent these voxels contribute positively in predicting pain intensity. This signature is developed with heat pain stimulation on volar surface of the left inner forearm in a healthy dataset (*N* = 20), and it can track the intensity of somatic pain in a new dataset (Wager *et al*., 2013). The NPS was obtained shared by the original authors by request (https://github.com/canlab/).

Two different versions of the VPS have been developed which we refer to here as VPS-Krishnan (Krishnan *et al*., 2016) and VPS-Zhou (Zhou *et al*., 2020). These are multivariate brain models designed to make predictions of vicarious pain. Similar to NPS, they are brain maps that include weights in each voxel albeit from the whole brain (rather than regions of interest). VPS-Krishnan and VPS -Zhou (applying their generalized classifier) were both obtained from https://github.com/canlab.

For each fMRI beta map, we computed the dot product of the beta values with the relevant weight map (NPS and VPS). This yields a continuous signature response value (SRV) for each participant and for each condition (see the formula below). This dot product was computed using the publicly available script from: https://github.com/canlab/.


$${\vec \beta _{map}} \cdot {\vec w_{map}}\mathop \to \limits^{yields} \,SRV$$

In effect, this reduces a pattern of multivariate data to a single numerical value (per participant, per condition) which can be analysed in a conventional way (e.g. *t*-tests, ANOVA). Our resulting data tended to be highly variable across participants and with significant outliers (note the large error bars for the SRVs in the figures below). As such, they were generally analysed non-parametrically via Wilcoxon tests (some data from VPS-Zhou were suitable for a parametric analysis). In effect, this is a forced-choice test where one is determining whether the SRV is numerically greater for the pain condition relative to the no-pain condition, noting that ‘Forced-choice tests are particularly suitable for fMRI because they do not compare the signature response with a threshold that is fixed across persons. Therefore, they do not require people to use the pain-reporting scale in the same way, and they do not require the scale of fMRI activity to be the same across scanners’ (Wager *et al*., 2013).

## Results

**Fig. 2.**
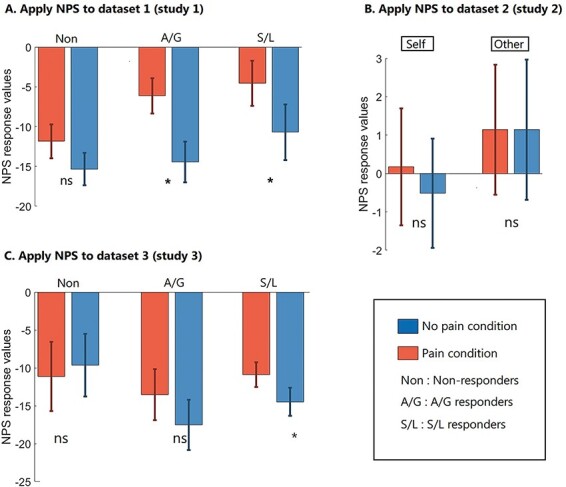
SRVs when applying the NPS to three independent datasets (* denotes *P* < 0.05).

**Fig. 3.**
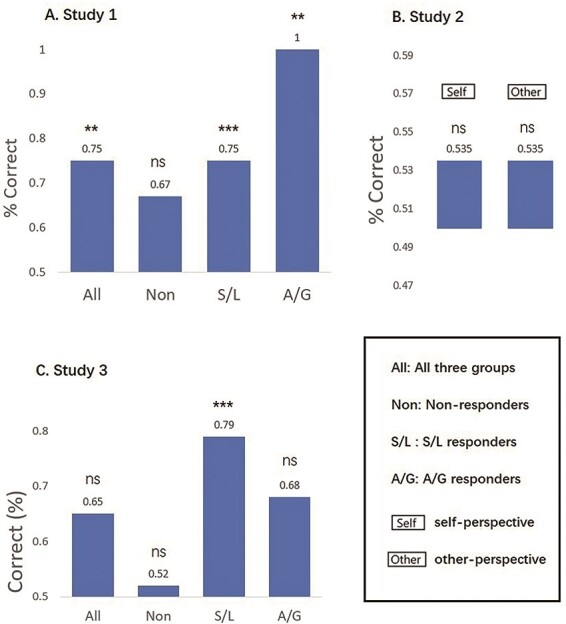
The results of the forced-choice comparison when applying NPS (* *P* < 0.05, ** *P* < 0.01, *** *P* < 0.001).

We report the results of the NPS first and then the two versions of the VPS. Figure 2 shows the SRVs when applying the NPS to the different conditions, and Figure 3 shows the results of the forced-choice comparison (i.e. determining if the SRV is numerically higher for pain relative to no-pain). For Dataset 1, we find that pain values are significantly higher than no-pain for the group as a whole (*P* = 0.001), and separately for S/L responders (*P* = 0.050) and A/G responders (*P* = 0.018). Non-responders fail to reach significance (*P* = 0.062). In Dataset 2, we find no significant differences between pain and no-pain in either the self-perspective (*P* = 0.164) or the other-perspective (*P* = 0.292) conditions. Recall that we do not know the vicarious pain status of this sample, although we assume most will be non-responders. In Dataset 3, we find that pain values are significantly higher than no-pain for the group as a whole (*P* = 0.009), but this is primarily driven by a significant difference in the S/L group (*P* = 0.016) and a non-significant trend in the A/G group (*P* = 0.053) with no significant difference in the non-responders (*P* = 0.968).

Combining the results of Dataset 1 and Dataset 3 and collapsing the two responder groups, we find that overall pain classification is higher for the responders (78.9%, 45/57) than non-responders (58.1%, 25/43) and this difference is significant [χ^2^(1) = 5.053, *P* = 0.025]. In summary, we find that a biomarker for physical pain can—in some circumstance—discriminate using forced-choice whether a person is viewing images of pain *vs* no-pain (in the absence of any physical pain). This result tends to be greater for vicarious pain responders.

**Fig. 4.**
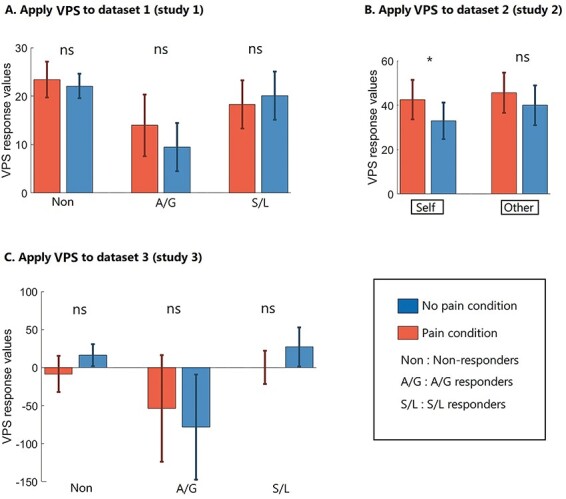
SRVs when applying VPS-Krishnan to three independent datasets (* *P* < 0.05).

**Fig. 5.**
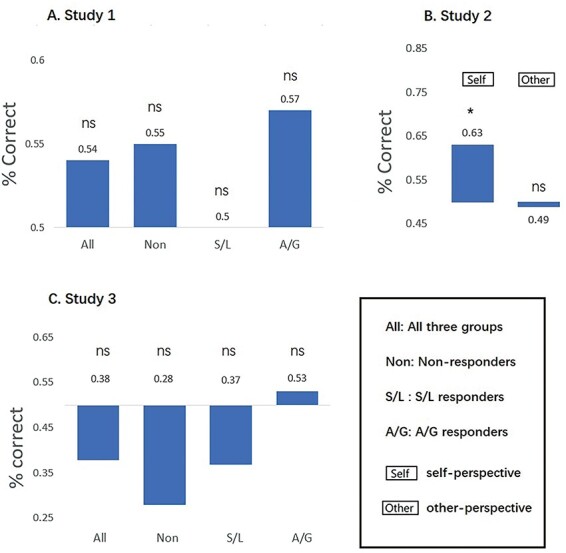
The results of the forced-choice comparison when applying VPS-Krishnan (* *P* < 0.05).

Figure 4 shows the SRVs when applying VPS-Krishnan to the different conditions, and Figure 5 shows the results of the forced-choice comparison (i.e. determining if the SRV is numerically higher for pain relative to no-pain). Here, the pattern is very different to the NPS. There is only one significant result. In Dataset 2 (Rae *et al*., 2020), we find that the self-perspective condition produces higher values for pain relative to no-pain (*P* = 0.048) with an accuracy level of 60.6% (43/71). Recall that this is a similar condition to the one used to create the VPS, in which participants take the perspective of the person depicted in the picture and then rate the imagined pain level (Krishnan *et al*., 2016). The self *vs* other interaction can be computed as difference of differences (i.e. a Wilcoxon test on pairs of differences), but this is not significant (z = 0.475, *P* = 0.635).

**Fig. 6.**
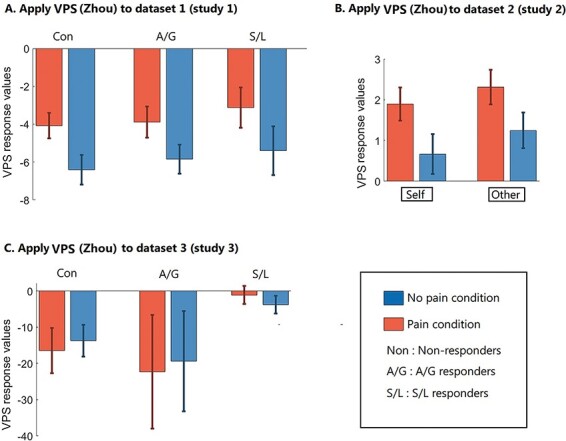
SRVs when applying VPS-Zhou to three independent datasets (* *P* < 0.05).

**Fig. 7.**
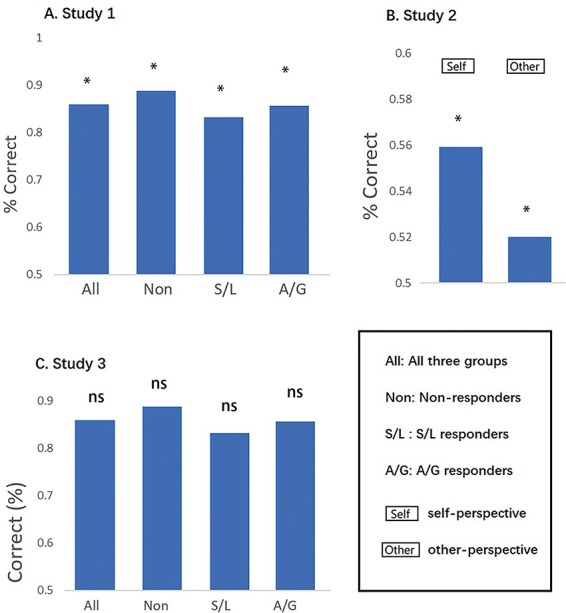
The results of the forced-choice comparison when applying VPS-Zhou (* *P* < 0.05).

Figure 6 shows the SRVs when applying VPS-Zhou to the different conditions, and Figure 7 shows the results of the forced-choice comparison. Overall, this has some success in distinguishing images of pain *vs* no-pain. Datasets 1 and 2 were analysed parametrically, whereas Dataset 3 contained significant outliers and was analysed non-parametrically. For Dataset 1, there was a main effect of pain *vs* no-pain [F(1,34) = 35.004, *P* < 0.001, η^2^ = 0.507] but no group main effect [F(2,34) = 0.351, *P* = 0.706, η^2^ = 0.020] or interaction [F(2,34) = 0.084, *P* = 0.920, η^2^ = 0.005]. Similarly for Dataset 2, there was a main effect of pain *vs* no-pain [F(1,70) = 45.722, *P* < 0.001, η^2^ =0.395] but no difference relating to self-other perspective [F(1,70) = 3.228, *P* = 0.077, η^2^ =0.044] and no interaction [F(1,70) = 0.404, *P* = 0.527, η^2^ =0.006]. For Dataset 3, performance was not significantly different from chance (although the S/L group showed a numerical trend of a similar magnitude to that observed in the other Datasets).

### Which regions of the NPS drive the group differences?

The NPS is comprised of some voxels that are positively weighted (i.e. indicative of physical pain) and others that are negatively weighted (i.e. contra-indicative of physical pain). Our group difference for the NPS could be driven by more positive beta values in positively weighted voxels or less negative beta values in negatively weighted voxels (or some intermediate combination). Another possibility is that there is lower data quality in the responders which may push the NPS score towards zero for theoretically uninteresting reasons.

**Fig. 8.**
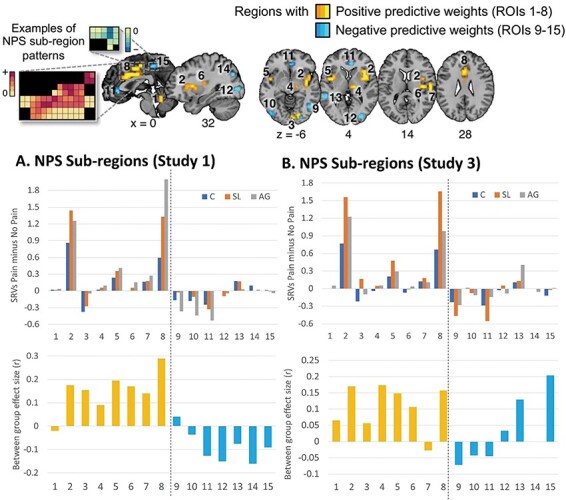
The NPS consists of 15 sub-regions some of which contain positive predictive weights (ROIs 1–8) and others contain negative predictive weights (ROIs 9–15). The top panel show mean SRVs (pain minus no-pain) across the 15 regions for the three groups, and the bottom panel shows the between group effect sizes across the regions (for responder > non-responder).

The NPS biomarker enables researchers to compute SRVs for 15 regions of interest (ROIs), 8 of which are overall positively weighted and 7 of which are negatively weighted (Wager *et al*., 2013b). SRVs were calculated separately for each of these 15 ROIs and for pain and no-pain beta maps (collapsing across hands and feet). Figure 8 shows the SRV differences contrasting pain minus no-pain for the three groups across the 15 regions for both Datasets 1 and 3 (i.e. where we observed differences at the level of the whole signature). The pattern is generally similar across both studies. Across all three groups, the largest numerical differences (pain > no-pain) were obtained for NPS sub-region 2 (right anterior insula) and sub-region 8 (mid-cingulate cortex). Between group differences were analysed by collapsing the responders into a single group (SL and AG) and comparing against non-responders. Due to violations of normality, they were analysed non-parametrically (Mann-Whitney U). No differences were statistically significant (at *P* < 0.05 uncorrected). Pooling data (SRVs for pain—no-pain) across these studies yielded a significant group difference (responder > no-responder) in mid-cingulate cortex (region 8) (*P* = 0.037). Effect sizes were computed for descriptive purposes, here using a non-parametric analogue of Cohen’s d recommended by Field (2005) in which the z-value computed in the non-parametric test is converted to r (z = r/√N) which has conventional interpretations: r > 0.1 being small, r > 0.3 being medium and r > 0.5 being large. Across both studies, the largest between-group effects were found in the positively weighted NPS regions including the mid-cingulate cortex (region 8), right and left anterior insula (regions 2 and 5) and right posterior insula (region 6). In all cases, the effect sizes were in the small (0.1 < r < 0.3) range. The SRVs for the negatively weighted regions of the NPS were numerically smaller (across all groups) and the between-group differences were less consistent across the two studies. Importantly, there is no evidence to suggest lower data quality in responders: if anything, their signals (pain *vs* no-pain) are stronger which is consistent with the finding that it is easier to distinguish images of pain from no-pain in that group via the overall NPS.

## Discussion

Across three datasets (two published, one new), we addressed the questions: can a biomarker developed for physical pain be used to distinguish vicarious pain (observing images of pain *vs* no-pain) and, more generally, do physical and vicarious pain share neural resources. To do so, we took advantage of existing multivariate weighted maps that have previously been shown to be sensitive to physical pain (the NPS) and vicarious pain (VPS, two versions). We also took an individual differences approach based on the observation that some people within the general population—as many as a quarter—report pain when they see others in pain (termed vicarious pain responders) (Osborn and Derbyshire, 2010; Grice-Jackson *et al*., 2017a). Here, the prediction was that shared representations between physical and vicarious pain may either be greater in these individuals or, possibly, unique to these individuals. We find positive evidence for the former prediction (greater in responders), but the study is not sensitive enough to definitively address the latter prediction (unique to responders).

### Does physical pain and vicarious pain have shared representations?

For the results of NPS, the results support our hypothesis that physical pain and vicarious pain have shared representations (to some degree) because a biomarker developed for physical pain can distinguish between viewing images of pain *vs* no-pain. The NPS response values of pain conditions are significantly higher than that of no pain conditions in responder groups, but not in controls. The results of forced-choice comparison show that NPS can predict pain/no-pain labels significantly above chance only in vicarious pain responders.

The results don’t speak against the general idea that the NPS may be a useful brain-based biomarker of pain but, instead, speaks to the familiar problem of defining ‘real pain’ given its multi-faceted nature and known dissociations (in both directions) between pain experiences and physical injury (Raja *et al*., 2020). This latter consensus paper proposed the definition: ‘An unpleasant sensory and emotional experience associated with, or resembling that associated with, actual or potential tissue damage’. It is well-accepted that pain experiences can occur in the absence of ongoing injury (e.g. phantom pain, Flor *et al*., 2006) and it is entirely conceivable that individual differences in vicarious pain (in response to seeing pain in others) constitute an analogous example. We note, however, that these experiences are subjectively far weaker and elicited via a different mechanism (presumably an internal simulation of pain) (Fitzgibbon *et al*., 2010). When asked to rate their vicarious pain experiences on a 0–10 scale (0 being no pain, and 10 being intense pain), the mean in vicarious pain responders is typically ∼3 (Ward and Li, 2022). Vicarious pain need not be excruciating or debilitating but it is sufficient for responders to endorse the term ‘pain’.

Previous research has distinguished between two types of vicarious pain responders. For example, those with the S/L sensory-localized profile show differences in the rubber-hand illusion (Botan *et al*., 2021b) and those with the A/G affective-general profile are more likely to report blood-injury phobic symptoms (Botan *et al*., 2021a). However, in many other respects, they are similar to each other (but different to non-responders): such as questionnaire measures of social and emotion functioning (Botan *et al*., 2018) and voxel-based morphometry (Grice-Jackson *et al*., 2017a). In the present research (Datasets 1 and 3), we show similar patterns for both of these groups although not always reaching the significance threshold due to small sample sizes. There may be differences between the groups that were too small to detect but, overall, the evidence suggests that vicarious pain responders are different from non-responders in terms of their NPS response.

### Does the VPS distinguish between images of pain and no-pain?

We considered three different datasets that used the same images of hands and feet in painful and non-painful contexts that have been used extensively elsewhere, including in the development of the whole-brain version of the VPS by Krishnan *et al*. (2016). A second version of the VPS was created from a similar set of images but additionally considered facial expressions and focused more on specific brain regions such as the insula (Zhou *et al*., 2020). We predicted that applying the VPS(s) would enable us to discriminate pain from no-pain fMRI beta maps in other studies using those images. A recent study comparing these signatures found only VPS-Zhou to be sensitive to individual difference (in healthcare training), although both VPS signatures performed well overall (Corradi-Dell’Acqua *et al*., 2023). In this study, VPS-Zhou was a good predictor of viewing images of pain *vs* no-pain (across all conditions/groups of Datasets 1 and 2) whereas VPS-Krishnan performed far worse (reaching significance only for the self-perspective in Dataset 2). We found no evidence that differences in either VPS mapped on to group differences in vicarious pain.

Our findings raise questions about what the VPS is actually sensitive to. VPS-Krishnan was created by asking participants to rate the pain intensity of the person depicted in the image, which is very similar to the self-condition of Rae *et al*. (2020). Arguably, one might claim that this is a task of perspective taking and it would appear to require attending to other-related information and suppressing one’s own responses to the image (Bird and Viding, 2014). As such, it is potentially different from vicarious pain as a form of other-to-self contagion (e.g. as reported by vicarious pain responders). Notably, the VPS did not have strong positive weights in the pain matrix (e.g. insula, cingulate, somatosensory cortices) but in regions such as the temporo-parietal junction involved in perspective taking and theory-of-mind (Jackson *et al*., 2006a; van der Heiden *et al*., 2013) . That is, VPS-Krishnan may be somewhat task-specific rather than being a marker of vicarious pain per se.

There is one other study that used VPS-Krishnan to successfully discriminate physical pain to one’s self *vs* pain administered to their partner, observable via a mirror and a cue signal (Lopez-Sola *et al*., 2017). Here, the task was to rate the unpleasantness when the pain was personally experienced or just watched (e.g. ‘How unpleasant was seeing your partner in pain?’). But the main dependent variable was the difference in signature values (NPS—VPS) for physical pain and vicarious pain, rather than a contrast between vicarious pain and vicarious no-pain (as in the analyses considered here). The latter is considered a better test because the comparison is well matched for extraneous variables.

The newer VPS-Zhou, in contrast, does have stronger weights in regions of the pain matrix such as the insula and has been shown to be sensitive to physical pain and vicarious pain in other datasets (Corradi-Dell’Acqua *et al*., 2023).

## Conclusion

This study makes two main contributions. First, we provide a possible explanation to the contradictory findings on the relationships of physical pain and vicarious pain through a consideration of individual differences. Second, we assess the generalizability of biomarkers developed for physical pain and vicarious pain to novel datasets and show that both have utility.

## Data Availability

Dataset 1 is available at https://neurovault.org/collections/14965/ (comprising individual beta maps for pain and no pain). Dataset 2 is available at https://neurovault.org/collections/8400/ (comprising group-level beta maps for pain and no pain and self/other conditions). Dataset 3 is available at https://neurovault.org/collections/15030/ (comprising individual beta maps for pain and no pain, also divided into hands and feet). The relevant Matlab code is on github at https://github.com/willstrawson/pain.
